# Outcomes of two different polytetrafluoroethylene graft sizes in patients undergoing maintenance hemodialysis

**DOI:** 10.4103/0971-4065.59336

**Published:** 2009-10

**Authors:** R. Afshar, S. Sanavi, S. Afshin-Majd, A. Davati

**Affiliations:** Department of Nephrology, Faculty of Medicine, Shahed University, Mustafa Khomeini Hospital, Tehran, Iran; 1 Department of Internal Medicine, Faculty of Medicine, Shahed University, Mustafa Khomeini Hospital, Tehran, Iran; 2 Department of Social Medicine, Faculty of Medicine, Shahed University, Mustafa Khomeini Hospital, Tehran, Iran

**Keywords:** Hemodialysis, polytetrafluoroethylene graft, vascular access

## Abstract

Arteriovenous access creation is mandatory for maintenance hemodialysis. If native fistula placement was not possible or failed, a prosthetic conduit would be the best substitute. The purpose of this prospective study was to compare outcomes of two different sizes of polytetrafluoroethylene (PTFE) grafts, in hemodialysis patients, at the Mustafa Khomeini Hospital in Iran. The study population consisted of 586 end-stage renal disease referrals for vascular access construction (January 2003 to January 2007) of which eventually 102 subjects were candidates for PTFE graft who were followed for one year. Data were collected by a questionnaire and analyzed using the SPSS, life table, Kaplan- Meier and Log-Rank tests. Out of 102 PTFE implantation candidates (mean value of age 51.7 ± 17.06 yrs), 56% were male and 44% female. PTFE grafts of 8 mm and 6 mm sizes were randomly placed in 57 and 45 subjects, with distribution of 83%, 12% and 5% in arm, forearm and thigh. The most underlying diseases were hypertension and diabetes. There was a significant difference in complication rates between patients with and without underlying diseases [42% *vs*. 10% (*P* = 0.03)]. One-year patency rates were 42.2% and 36.5% for 6 mm and 8 mm grafts and 28.2% *vs*. 52% in patients with and without underlying diseases respectively. Despite more complication frequency in 8 mm grafts, the patency and complication rates of two graft groups did not significantly differ. Hypertension and diabetes could have contributory roles in graft complication rate, which may be preventable. Non-tapered grafts of 6 mm and 8 mm sizes have not significant different outcomes. Further research is recommended with larger sample size and longer duration.

## Introduction

Hemodialysis requires access to blood vessels capable of providing rapid extracorporeal blood flow. These requirements are currently best met by arteriovenous fistulas (AVF). The goal of chronic vascular access is to provide repeated access to the circulation with minimal complications. Synthetic grafts are constructed by anastomosing a synthetic conduit, usually polytetrafluoroethylene (PTFE, also known as Gore-Tex), between an artery and vein. The conduit can be straight or looped and ranges between 4 to 8 mm in diameter. Grafts can be modified to be tapered at the arterial side, to decrease complications.[1,2] The 2006 K/DOQI work group recommends a graft either of synthetic or biologic material.[3] Common graft locations are straight forearm (radial artery to cephalic vein), looped forearm (brachial artery to cephalic vein), straight upper arm (brachial artery to axillary vein), or looped upper arm (axillary artery to axillary vein). The 2006 K/ DOQI work group prefers a forearm loop graft, preferable to a straight configuration.[3] Chronic hemodialysis access complications include thrombosis, infection, steal, aneurysms, venous hypertension, seromas, heart failure, and local bleeding. Thrombosis, infection, and seromas appear to occur more frequently with grafts than with fistulas.[4–7] The main complication of hemodialysis grafts is stenosis at the venous side due to intimal hyperplasia, leading to graft dysfunction and thrombosis.[8,9] The purpose of this prospective study was to compare outcomes of two different sizes; hemodialysis grafts of 6 mm and 8 mm diameters.

## Materials and Methods

The study population composed of 586 end-stage renal disease (ESRD) patients referred for vascular access construction, between January 2003 and January 2007, at the Mustafa Khomeini Hospital in Tehran, Iran. Patients were examined by an expert vascular surgeon. All participants were informed of study design and purposes and signed a consent form before undergoing surgical implantation. Out of 586 referrals, 102 patients (57 males, 45 females) were candidates for expanded PTFE (Gore-Tex) graft construction (either because of inappropriate veins or native fistula failure) who were followed at least for one year. The patients' follow-up was obtained from their dialysis centers by 1, 3, 6, 9 and 12 monthly requests. The graft sizes of 8 mm and 6 mm were implanted randomly in 57 and 45 subjects. The graft site was decided upon previous patients' surgical records and existence of proper vessels for graft implantation, so that 85, 12 and 5 grafts were placed in arm, forearm and thigh, respectively. Data including demographic information, underlying diseases, hemodialysis sessions frequency, hemodialysis duration, graft function and complications (thrombosis, infection, steal, aneurysms and bleeding) were collected by a questionnaire.

Primary patency rate (PR) was defined as normal graft function from the time of implantation until graft failure or malfunction requiring surgical correction. Early graft failure was declared as access abandonment less than 30 days after graft placement. All data analyses were carried out using the statistical software package SPSS, version 16 and survival distributions were plotted using the Kaplan-Meier method for graft survival (primary patency). Functional grafts on last follow-up examination which were discontinued for reasons other than failure, such as transplantation or death were censored in the life-table analysis. Log-Rank test was used to evaluate statistical differences in survival distribution between two groups. *P* value < 0.05 was considered statistically significant.

## Results

**Figure 1:**
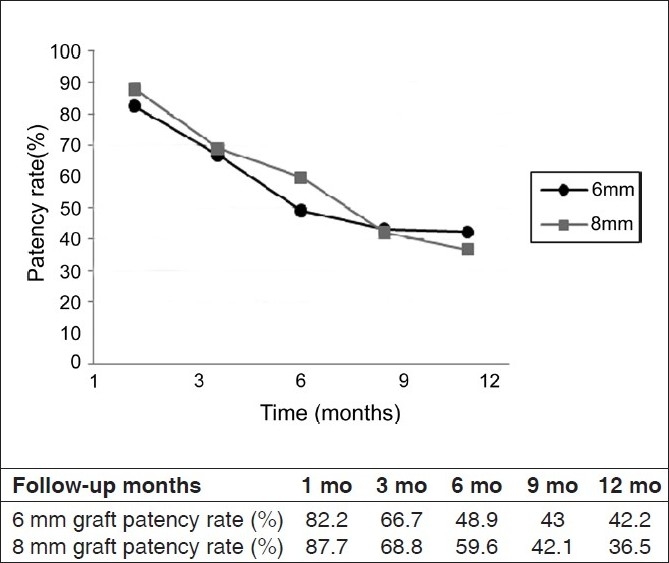
Primary patency rates for both 6 mm and 8 mm hemodialysis grafts during 12 month follow-up

The PTFE graft candidates, 56% male and 44% female, were between the ages of 15 to 91 (mean 51.7 ± 17.06) years [Table 1]. Hypertension, diabetes, and diabetes associated with hypertension were found in 27%, 14% and 24% of cases, while the underlying disease was unclear in 28% of patients [Table 2]. Hemodialysis frequency was from 1 to 3 sessions per week and each session lasts 3-4 hours. The mean duration of hemodialysis was 20 ± 1.5 months. There was no operative mortality in this study, but 11 patients died from serious complications of their renal disease during the follow-up period and 5 subjects underwent renal transplantation. Therefore, eventually 16 patients were excluded from the study. Complications including thrombosis, infection, infection associated with thrombosis, aneurysm and infected aneurysm were observed in 26%, 14%, 10%, 1% and 1% of patients respectively (overall complication rate = 52%). A significant difference in complication rate was found between patients with and without underlying diseases (*P* = 0.03). Despite more thrombosis occurrence in 8 mm grafts (34% *vs*. 18%), the overall complication rates in two grafts did not differ significantly (*P* = 0.07). The primary patency rates at one year were 42.2% *vs*. 36.5% for 6 mm and 8 mm grafts and 52% *vs*. 28.2% in patients without and with underlying diseases, respectively. There was no difference in patency rates, between 6 mm and 8 mm grafts (*P* = 0.1) [Figure 1]. In addition, the Spearman test showed that age, gender, frequency of dialysis sessions per week, hemodialysis duration had no relationship with patency and complication rates and complication type. Lower limb grafts had poorer survival than upper limb grafts (complication rate: 60%; PR: 100%, 40%, 20%, 20% and 0 at 1, 3, 6 and 12 months). Early failure was found in seven cases in each graft group (total 14%).

**Table 1:** Gender distribution in two different sizes graft groups

Gender	Males	Females
		
Gore-Tex size		
8 mm	n = 27	n = 30
6 mm	n = 30	n = 15
Total	57	45

N - Number of patients

**Table 2:** Underlying disease distribution in two different sizes graft groups

Gore-Tex size	8 mm	6 mm
		
Underlying diseases		
Hypertension	n = 16	n = 12
Diabetes mellitus	n = 9	n = 5
HTN and DM	n = 18	n = 6
Miscellaneous	n = 4	n = 3
None	n = 10	n = 19
Total	57	45

N - Number of patients, HTN - Hypertension, DM - Diabetes

## Discussion

Hemodialysis is the commonst technique for renal replacement therapy of ESRD patients throughout the world. On the other hand, maintaining patients on hemodialysis depends on a vascular access construction with low complications and long durability against needling. Preferably, an autologous AVF, either in the non-dominant wrist or elbow crease (upper extremities), is the best first choice for hemodialysis vascular access. However, native AVF creation may be impossible because of obliteration of major superficial veins by previous medical interventions.[10–12] In these situations, a prosthetic arteriovenous conduit is implanted. With respect to high cost of such grafts, it is important to know more about their optimal characteristics. The optimal graft diameter for hemodialysis is yet to be determined. It is recommended to implant no more than 4 mm graft size at the arterial side to avoid cardiovascular complications, but many surgeons implant 6 mm grafts in different anatomical locations.[13,14] Implantation of 8 mm grafts tapered to 4-5 mm at the arterial side has been recommended for upper arm dialysis grafts.[15,2] Based on large-bore graft advantages including easy needling and lower occurrence of mid-graft stenosis due to intimal hyperplasia, we preferred to implant 6 and non tapered 8 mm grafts particularly in upper arm position, because of improper veins and conditions in the forearm as recommended.[3]

Compared with other centers, the frequency of graft placement at the Mustafa Khomeini Hospital is high (17.3%), because it is one of referral centers of vascular surgery in Iran which admits difficult and complicated cases with multiple AVF failures. In comparison to other studies, primary patency rate at one year in our study was 40% *vs*. 58%.[12,16,17] In addition, we did not find any significant difference in complication and patency rates between two grafts as reported by Garcia-Pajares *et al*.[2] The differences in results among these studies may be attributed to following reasons: Using grafts with similar diameter throughout the length, without tapering at the arterial side which lowers complications;unfamiliarity of hemodialysis unit staff and patients with proper care of vascular grafts; and late referral of complicated patients for intervention. Our complication rates were similar to the reported studies except one.[2] Contrary to some studies,[18,19] we found significant relationship between hypertension-diabetes and complication rate which indirectly can impact on patency rate. Also, upper extremity grafts had higher one-year patency rate (40%) than lower limb (0) grafts, similar to other studies[11] and early graft failure did not occur in lower extremities. However, because of small sample size, these findings must be cautiously considered and one-year patency rate up to 60% for lower limb grafts have also been reported.[20,21] Early failure was found in seven cases in 15% and 12% in the 6 mm and 8 mm grafts, which indicated that diameter tapering at the arterial side did not contribute to early graft dysfunction.[2]

## Conclusion

We concluded that without considering some changes in 6 mm and 8 mm grafts, including arterial side diameter tapering, the outcomes of these grafts did not differ, substantially. It seems there is no advantage for 6 mm grafts over 8 mm grafts, except in more thrombosis occurrence in 8 mm grafts. Hypertension and diabetes may have an undesirable influence on hemodialysis grafts outcomes. The effects of hypertension and diabetes on vascular grafts outcomes can be attributed to atherosclerosis and alterations in blood pressure due to antihypertensive drugs in hypertensive patients or vascular structural disorders and hypotension due to autonomous nervous system dysfunction (hypo perfusion) in diabetics.
